# Tibialis Posterior Tendon Dysfunction Due to Syndesmotic Fixation Using Non-absorbable Suture Button Device Following Fracture-Dislocation of the Ankle: A Case Report

**DOI:** 10.7759/cureus.72442

**Published:** 2024-10-26

**Authors:** Aysha Rajeev, Saurav Krishnan, George Koshy, Mintu Mariam Baby, Kailash Devalia

**Affiliations:** 1 Trauma and Orthopaedics, Gateshead Health Foundation NHS Trust, Gateshead, GBR; 2 General Medicine, Gateshead Health Foundation NHS Trust, Gateshead, GBR

**Keywords:** fracture dislocation of the ankle, suture button, syndesmosis injury, tibialis anterior tendon transfer, tibialis posterior tendon dysfunction

## Abstract

Fracture-dislocation of the ankle is a common lower limb injury, often associated with syndesmotic damage. While CT and MRI scans are more sensitive than plain radiographs in diagnosing syndesmotic injuries, treatment typically involves either static stabilization using metallic screws or dynamic stabilization with a suture button device. The primary advantage of dynamic stabilization is that it eliminates the need for a second surgery to remove screws. However, chronic syndesmotic injuries can lead to significant morbidity, including pain and impaired function. In patients with inflammatory conditions, such as rheumatoid arthritis, dynamic stabilization may result in loosening and migration of the suture button, potentially causing tendon degeneration and rupture.

We present the case of a 63-year-old woman with rheumatoid arthritis who sustained a fracture-dislocation of the ankle. She was treated with open reduction and internal fixation, using a plate, screws, and a suture button device for syndesmotic stabilization. Postoperatively, she developed a plano-valgus deformity due to migration of the suture button into the tibialis posterior tendon and degenerative arthritis of the syndesmosis joint.

## Introduction

The incidence of syndesmosis injury in ankle fractures ranges from 20% to 45% [1]. In Weber B fractures, syndesmosis injury is associated with 17% to 39% of cases [2], while in Weber C fractures, this association increases to 80% [3]. The tibiofibular syndesmosis is a fibrous joint that articulates the fibula with the tibia and is stabilized by four ligaments: the anterior inferior tibiofibular ligament (AITFL), the interosseous ligament (IOL), the transverse ligament (TL), and the posterior inferior tibiofibular ligament (PITFL). These ligaments prevent excessive fibular movement in various directions, including anterior-posterior and lateral translation, as well as internal and external rotation. During injury, excessive external rotation of the ankle in dorsiflexion, combined with foot adduction or abduction, can cause widening of the fibula relative to the tibia at the ankle joint, resulting in disruption of the syndesmotic ligaments and secondary talar shift.

Treatment of syndesmotic injuries can be either static fixation with screws or dynamic fixation using a non-absorbable button device (tightrope) [4]. Static screw fixation eliminates motion at the distal tibiofibular joint, but screw breakage occurs in 7% to 29% of cases [5]. Willmott et al. reported soft tissue irritation and swelling associated with suture button devices [6]. There are also case reports of tibialis posterior tendon entrapment and rupture in the context of acute syndesmosis injuries [7,8]. However, no cases of tibialis posterior tendon dysfunction causing plano-valgus deformity following suture button fixation have been reported in the literature.

We present a rare case of fracture-dislocation of the ankle with syndesmosis injury, treated with a suture button device, that was complicated by tibialis posterior tendon dysfunction. This required tendon reconstruction and arthrodesis of the distal tibiofibular joint. This case report underscores the importance of precise placement of the suture button device and achieving congruent reduction of the syndesmosis joint. Additionally, it highlights that the use of a suture button device in patients with inflammatory joint disease or osteoporosis may increase the risk of device loosening. This can lead to migration of the button into the tendon sheaths, causing tendon dysfunction and loss of syndesmosis reduction, ultimately contributing to the development of secondary osteoarthritis.

## Case presentation

**Figure 1:**
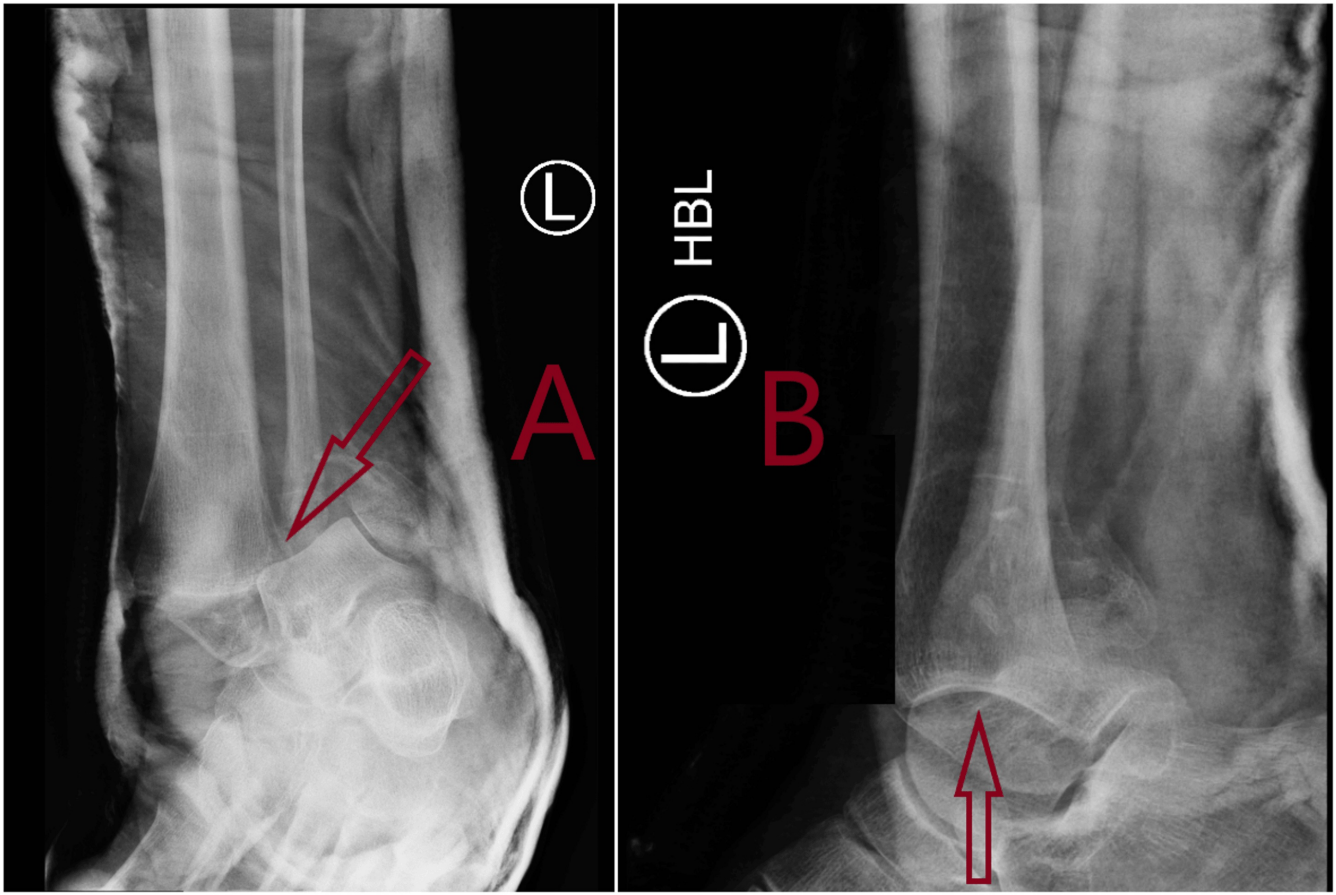
Plain X-rays showing pre-reduction lateral and oblique views of fracture dislocation of left ankle in Accident & Emergency. A: Anteroposterior (AP) view of fracture dislocation of left ankle. Arrow shows fracture dislocation with syndesmosis injury. B: Lateral view of fracture dislocation of left ankle. Arrow shows fracture dislocation of ankle.

**Figure 2:**
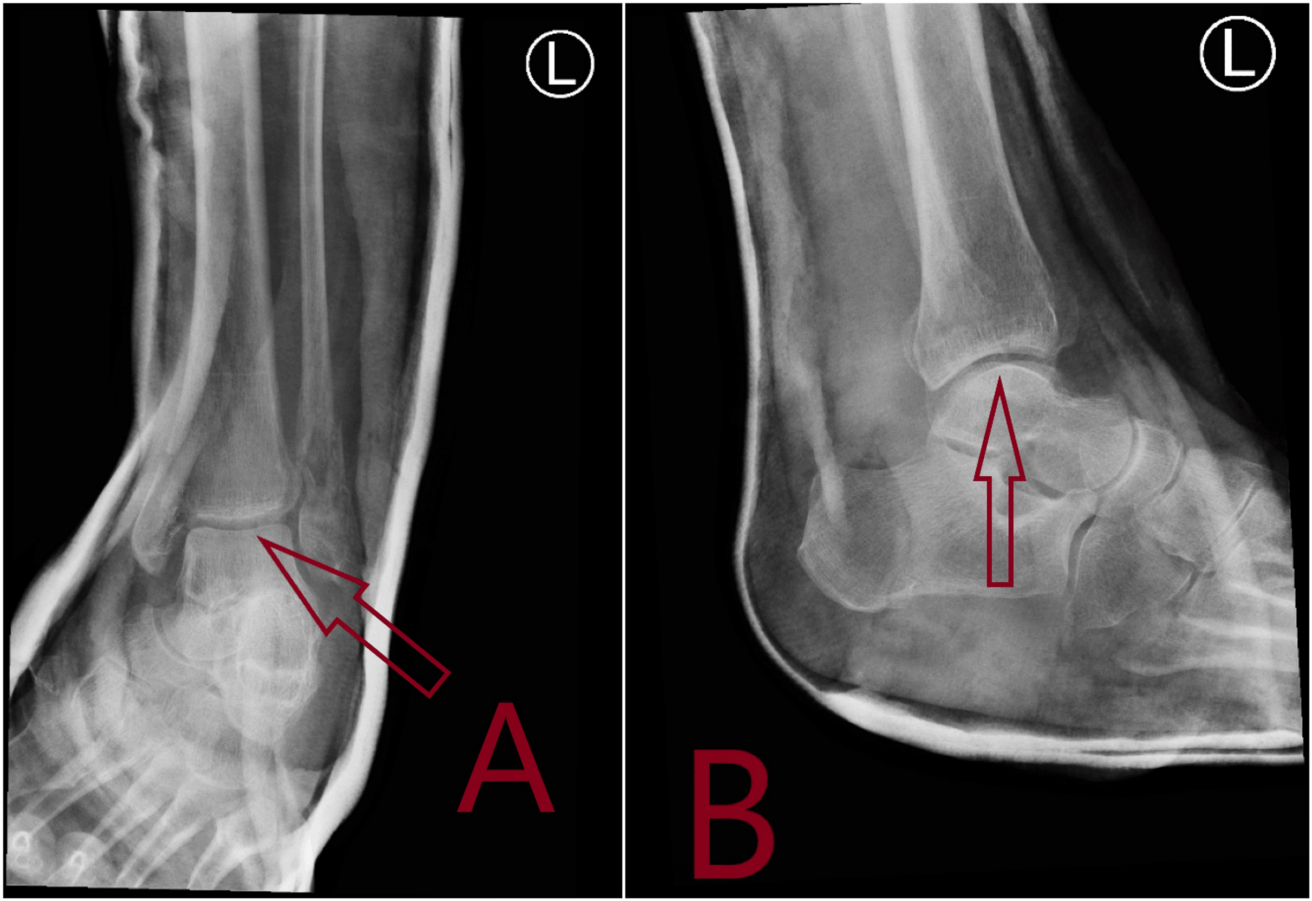
Plain X-rays showing post-reduction of fracture dislocation of left ankle in Accident & Emergency. A: Anteroposterior (AP) view X-ray with arrow showing congruent reduction of ankle joint. B: Lateral view X-ray with arrow showing congruent reduction of ankle joint.

**Figure 3:**
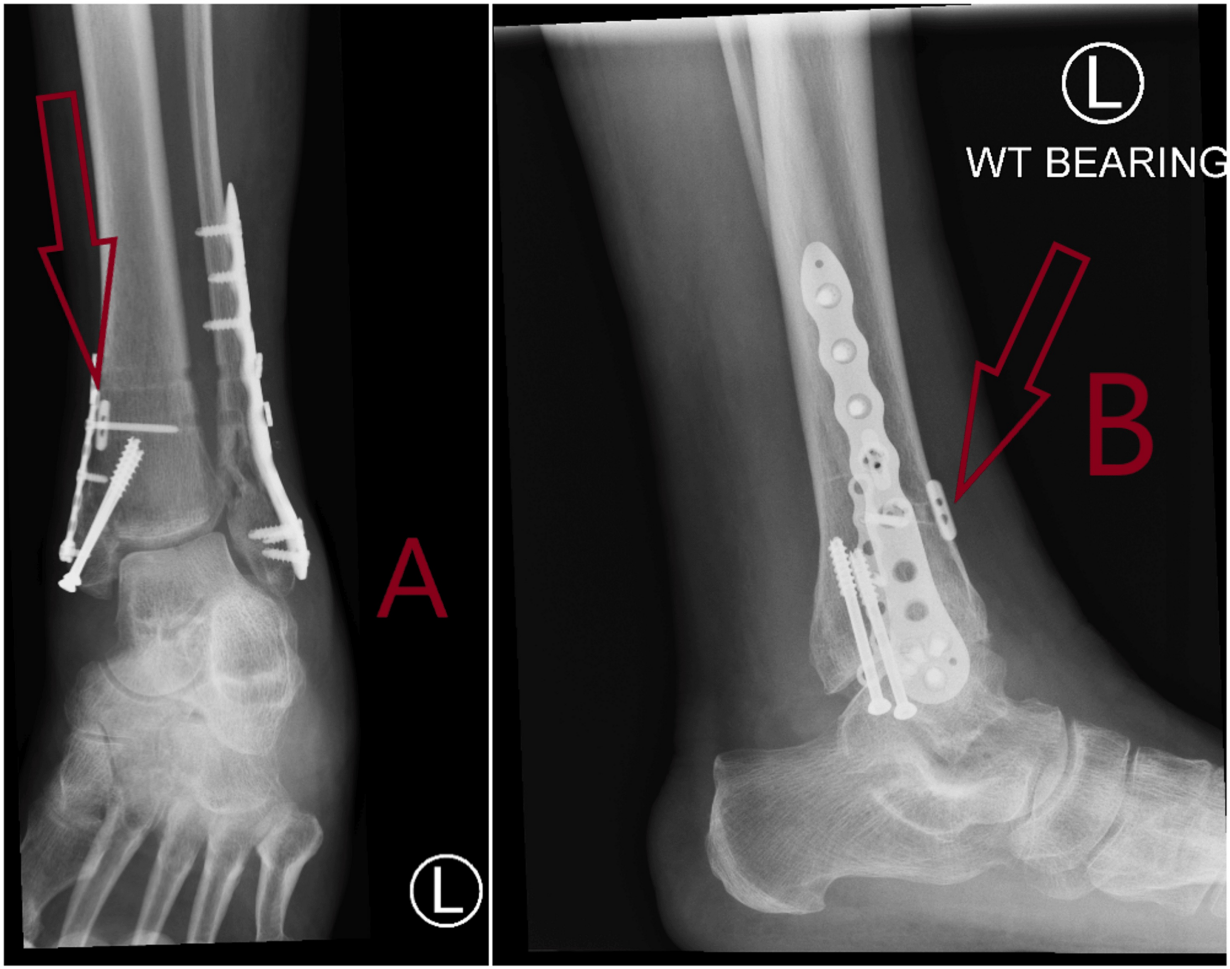
Plain radiographs at six weeks showing fracture healing with suture button device in situ. A: Anteroposterior (AP) view at six weeks showing fracture healing with arrow showing suture button device in situ. B: Lateral view at six weeks showing fracture healing with arrow showing suture button device in situ.

A 63-year-old woman was admitted to our unit following a fall from a standing height onto her left side, resulting in a fracture dislocation of the left ankle, which was reduced in the Emergency Department (Figures 1, 2). She had a medical history of rheumatoid arthritis and anxiety. After discussing the treatment options, the patient underwent open reduction and internal fixation with stabilization of the syndesmosis using two suture button devices (Figure 3). Her postoperative recovery was uneventful. She was treated in a below-knee non-weight-bearing cast and, after six weeks, the cast was removed. Rehabilitation, including passive and active movements of the ankle joint and progression from partial to full weight bearing, was initiated.

Five months postoperatively, the patient reported persistent discomfort on the medial aspect of her left ankle, which was interfering with her ability to resume routine activities. On examination, the surgical scars on the medial and lateral aspects of the ankle had healed satisfactorily. There was no tenderness on the lateral aspect, but she exhibited tenderness along the course of the tibialis posterior tendon and a sensitive medial scar. The ankle had a reasonable range of motion, and she could bear weight on the left leg, although the foot was in a plano-valgus position with loss of the medial arch.

**Figure 4:**
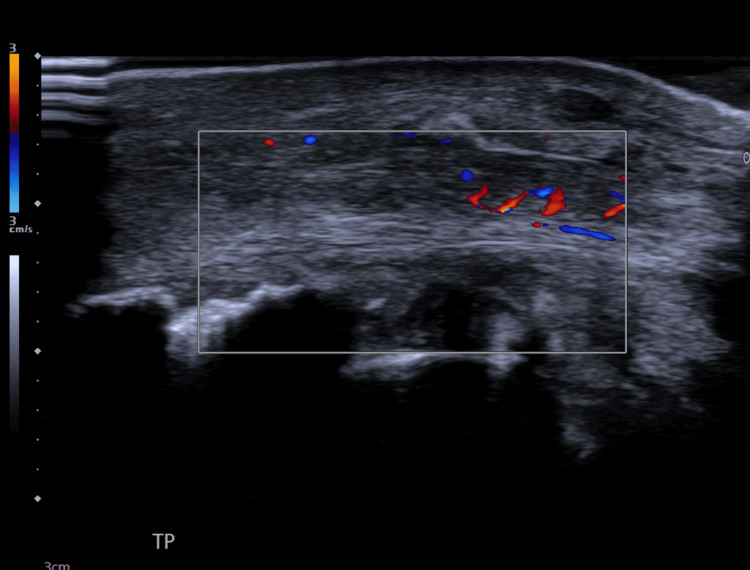
Ultrasound scan film showing partial tear and degenerative changes of tibialis posterior tendon.

**Figure 5:**
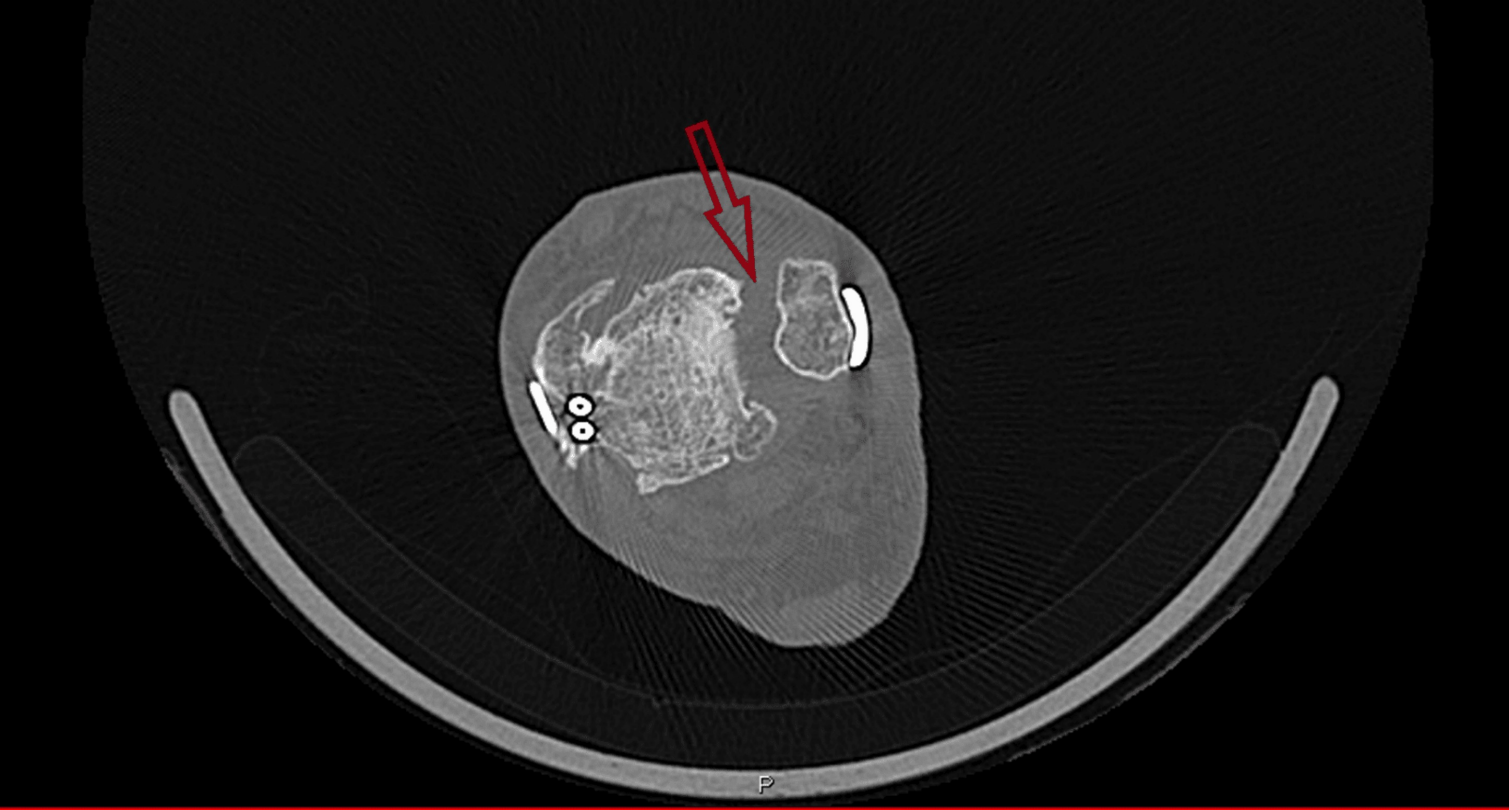
Axial section of CT ankle showing syndesmosis joint maligned and degenerate. Arrow showing syndesmosis joint maligned and degenerate.

An ultrasound scan revealed generalized enlargement of the tibialis posterior tendon with grade 3 tendinopathy, significant intrasubstance neovascularity, and partial tears, although the tendon remained intact. There was also moderate effusion within the tendon sheath (Figure 4). A CT scan showed malalignment and degeneration of the syndesmosis (Figure 5).

After discussing the findings with the patient, a staged approach to address the plano-valgus deformity, tibialis posterior tendon tear, and degenerative changes in the syndesmosis joint was planned.

Stage one involved the removal of the suture button device, medial plate, and screws. Intraoperatively, one of the suture buttons was found to have loosened and migrated posteriorly into the tibialis posterior tendon sheath, causing partial tearing and degeneration of the tendon. The lateral plate and screws were left in place.

**Figure 6:**
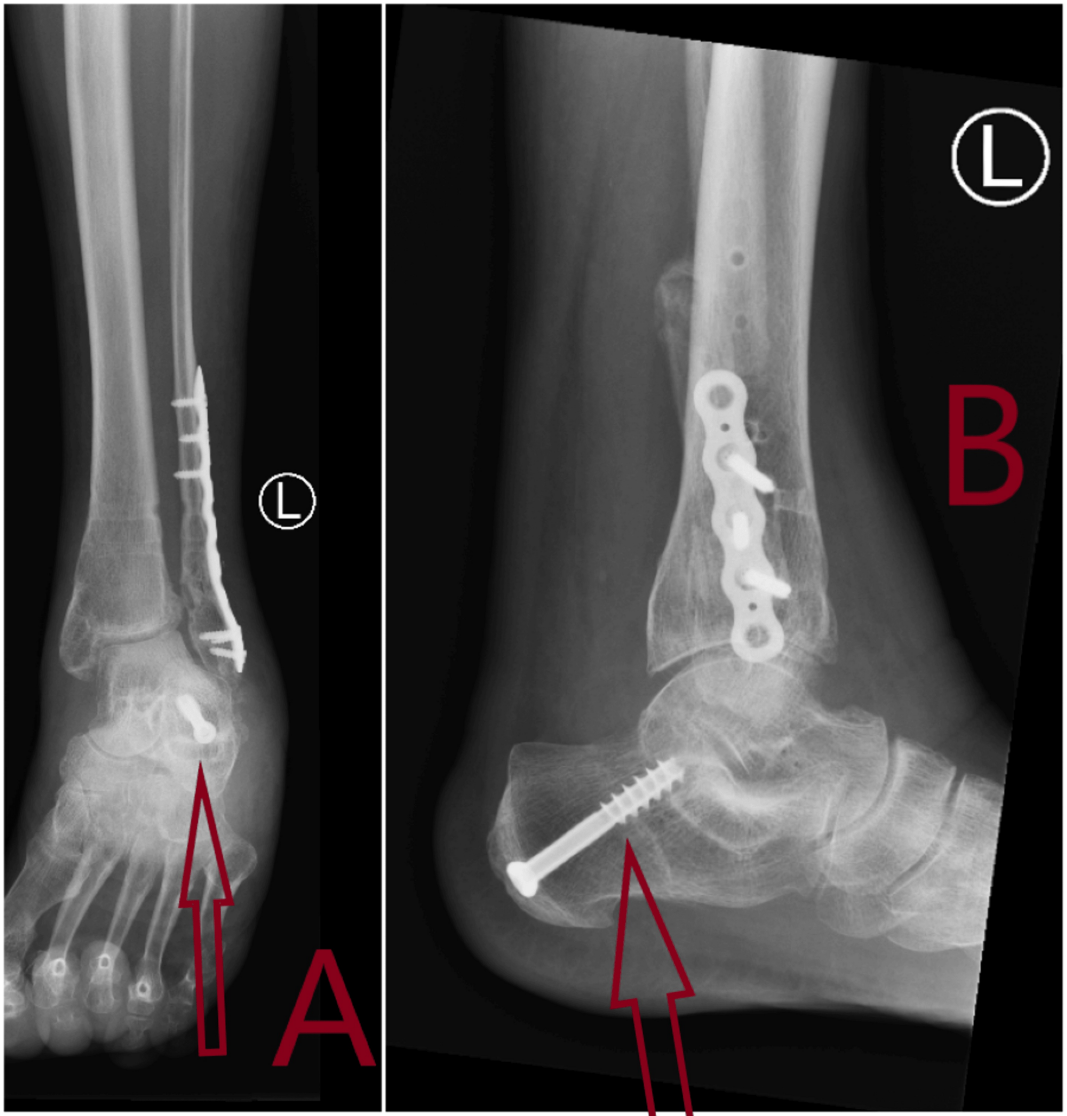
Post-operative radiographs showing healed calcaneal osteotomy and correction of plano valgus deformity. A: Anteroposterior (AP) post-operative radiograph with arrow showing correction of plano valgus deformity. B: Lateral post-operative radiograph with arrow showing healed calcaneal osteotomy.

Stage two involved debridement and reconstruction of the tibialis posterior tendon using a split tibialis anterior tendon transfer, along with a calcaneal osteotomy to correct the plano-valgus deformity (Figure 6).

Stage three included syndesmosis joint arthrodesis with iliac crest bone grafting and fibular osteotomy to restore fibular length. At six weeks, radiological evidence of bony union was observed, and the patient was permitted a full range of motion and weight-bearing.

**Figure 7:**
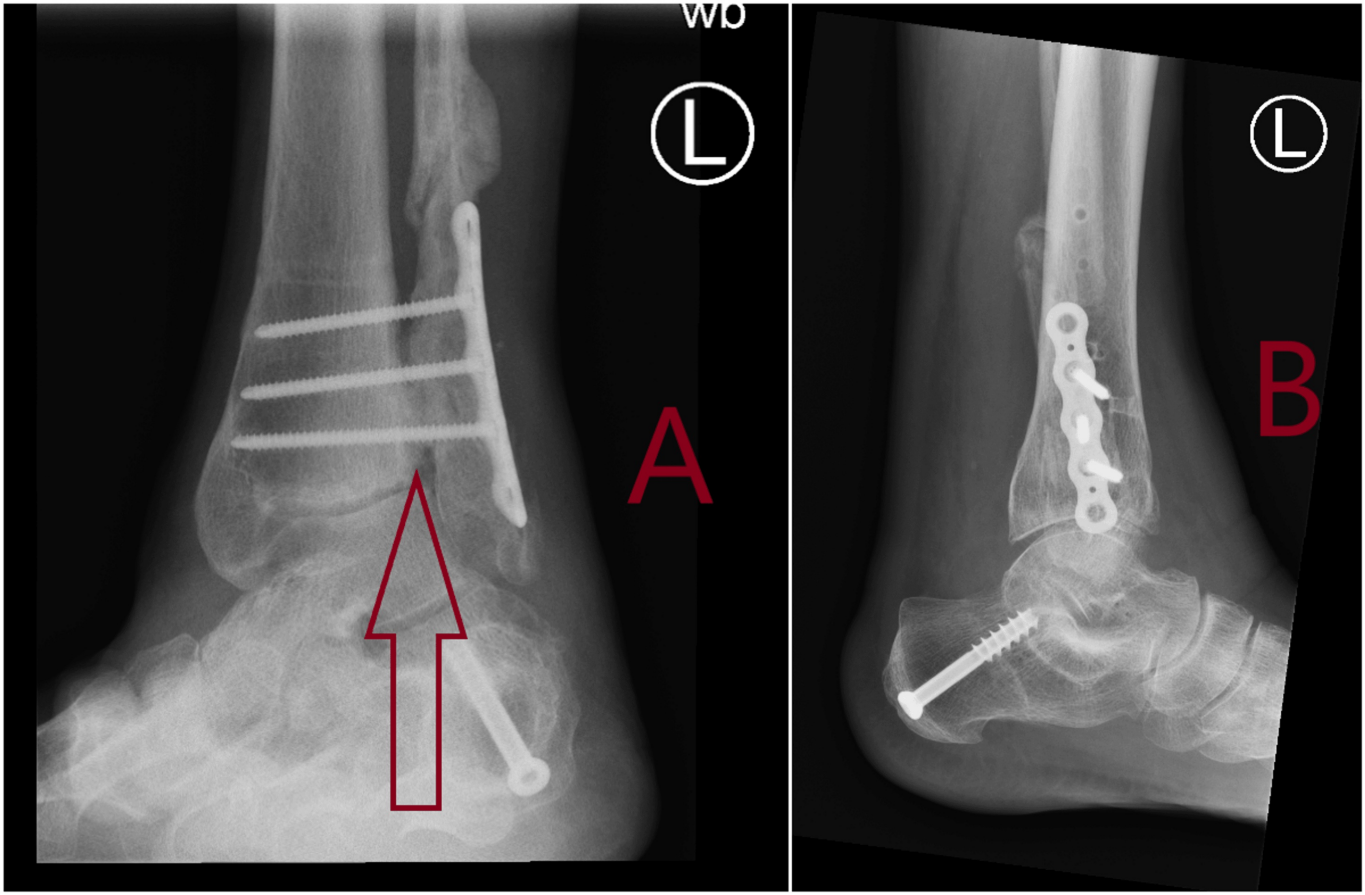
Radiographs at six months showing fusion of the syndesmosis joint. A: Anteroposterior (AP) view at six months with arrow showing fusion of the syndesmosis joint. B: Lateral view at six months.

At the final follow-up, six months after syndesmosis fusion, the patient had achieved solid bony union, a good range of ankle movement, and full weight-bearing capability (Figures 7A, 7B). Patient-reported outcomes were assessed using the Manchester Oxford Foot Questionnaire (MOxFQ). This questionnaire evaluates various aspects of foot and ankle function, including pain at rest, pain during walking and daily activities, night pain, walking distance, walking on uneven surfaces, and difficulty wearing appropriate footwear. In this case, the patient's MOxFQ score showed significant improvement, decreasing from 64 preoperatively to 31 at the final follow-up.

## Discussion

The distal tibiofibular syndesmosis is crucial for maintaining the congruency of the ankle mortise and supporting joint stability [9,10]. Syndesmotic injuries can occur in isolation or in conjunction with malleolar fractures [11]. These injuries are often caused by rotational forces, such as those involved in pronation-external rotation, pronation-abduction fractures, or, less frequently, supination-external rotation fractures [12]. Maisonneuve fractures, which involve a high fibular fracture due to rotational ankle injury, are well-known for having a high incidence of syndesmotic injuries [13].

Patients with isolated syndesmosis injuries typically present with pain at rest, swelling, instability, and difficulty in weight bearing [14]. Specific tests for syndesmosis injury include the squeeze test, where pain is elicited by compressing the tibia and fibula at the midpoint of the calf [15], the Frick test, which involves applying external rotation to the foot and ankle with the knee flexed at 90° [16], and the Cotton test, in which medial and lateral forces are applied to the talus; increased medial-lateral plane translation indicates a positive result [17].

Imaging for syndesmosis injuries begins with plain radiographs, including anteroposterior (AP), lateral, and mortise views, which can evaluate diastasis of the distal tibiofibular joint. Key measurements include the tibiofibular clear space (normally ≤6 mm), tibiofibular overlap (≥6 mm on AP view and ≥1 mm on mortise view), and medial clear space (<4 mm) [18]. In cases where syndesmotic injuries are associated with fractures, preoperative CT is beneficial for assessing fracture morphology, particularly the size and shape of posterior malleolar fractures, and for surgical planning [18]. MRI is useful in evaluating soft tissues, especially in acute and chronic isolated syndesmotic injuries [19]. In our case, CT and ultrasound were used to assess both the syndesmosis and tibialis posterior tendon for degeneration and tears, while MRI was not performed due to potential metal artifacts.

Treatment for syndesmosis disruption can involve either static or dynamic stabilization methods. Static fixation typically uses metallic or bioabsorbable screws, staples, syndesmosis hooks, or Kirschner wires [20]. Trans-syndesmotic screw fixation is a traditional and effective method for stabilizing the syndesmosis to allow ligament healing. Variables such as screw size, the number of cortices involved, and the position of the foot at the time of fixation do not significantly impact functional outcomes. However, complications such as screw loosening, breakage, discomfort due to over-compression, and the need for reoperation for screw removal, as well as the risk of late diastasis following early screw removal, are known issues [21].

The suture button device involves a No. 5 fiber-wire loop that can be tensioned and secured between two metallic endobuttons placed against the outer cortices of the tibia and fibula or fibular plate. It offers dynamic stabilization of the ankle mortise, reducing the need for removal and minimizing the risk of late diastasis [20]. However, complications including infection, skin irritation, granuloma formation, osteomyelitis, and osteolysis have been reported, which may necessitate device removal [22].

Cataldi et al. reported a case of complete tibialis posterior tendon rupture associated with syndesmosis injury, suggesting a novel mechanism of avulsion of the anterior inferior tibiofibular ligament without fibular fracture [8]. Sato et al. described a case of tibialis posterior tendon entrapment in the syndesmosis region following an ankle fracture-dislocation [7]. In our case, tibialis posterior tendon dysfunction occurred due to loosening and migration of the endobutton, which caused irritation and attrition of the tendon. The patient's rheumatoid arthritis may have contributed to the Grade 3 tendinopathic changes and partial tendon tears. A three-stage procedure was required to correct the tendon dysfunction, plano-valgus deformity, and secondary degenerative arthritis of the syndesmosis joint.

## Conclusions

Tibialis posterior tendon dysfunction following fixation with a suture button device for syndesmosis disruption is extremely rare. In cases of ankle fracture-dislocation with syndesmosis injury, the decision between static stabilization with metallic screws and dynamic fixation with a suture button device should be carefully considered. In patients with inflammatory joint diseases, such as rheumatoid arthritis, and those with osteoporosis, dynamic stabilization with a suture button device may increase the risk of endobutton loosening and migration. This can lead to soft tissue irritation, tendon degeneration, and potential ruptures.
